# Analytical investigation of soliton propagation in conformable fractional-order transmission line metamaterials

**DOI:** 10.1038/s41598-025-31060-8

**Published:** 2026-02-12

**Authors:** Ehab M. Almetwally, Mohammad A. Zayed, Sara I. Abo-Hashem, Rahma Sadat, Samah M. Mabrouk, Ahmed S. Rashed

**Affiliations:** 1 Department of Mathematics and Statistics, College of Science, Imam Mohammad Ibn Saud Islamic University (IMSIU), 11432 Riyadh, Saudi Arabia; 2 Department of Physics and Engineering Mathematics, Faculty of Engineering, Zagazig University, Zagazig, Egypt

**Keywords:** Conformable fractional derivative, Nonlinear transmission lines, Metamaterial waveguides, Nonlinear electric circuit, Applied mathematics, Electrical and electronic engineering

## Abstract

This research is devoted to analyze a conformable fractional-order model of nonlinear two-dimensional transmission line metamaterials. The conformable fractional derivative is utilized to broaden classical differentiation while maintaining essential aspects, including the chain rule. Transmission line metamaterials, as intentionally designed structures, offer a robust framework for examining wave transmission in nonlinear dispersive media. The suggested electrical model facilitates understanding the behavior of creation and transmission of solitons, examined via voltage wave dynamics in circuit-based representations including inductors and capacitors. Due to the electromagnetic characteristics, metamaterials possess considerable promise for use in microwave engineering, signal processing, and communications. Three analytical techniques are utilized to investigate the model’s dynamics: the sine–Gordon expansion method, the fractional sub-equation approach, and the tanh method. Each approach was implemented within the fractional-order interval of the derivative parameter, 0 < α ≤ 1. The derived solutions demonstrated both singular and multiple soliton configurations, essential for the development of sophisticated waveguides and the improvement of signal transmission in telecommunications. The comparison research revealed that all three strategies effectively generated soliton solutions; however, the sine–Gordon expansion method was more beneficial, as it produced more generalized solution forms. This underscores its adaptability and wider applicability in the modeling and construction of fractional-order metamaterial systems.

## Introduction

Conformable fractional-order model of transmission line metamaterials has made wave propagation in engineered media a major focus. Artificially structured materials that can host electromagnetic properties not present in natural materials are called metamaterials. Such properties are imparted to these materials by their artificially engineered structural properties, which enable a certain range of control over wave behavior, whether acoustic or electromagnetic waves. This flexibility in properties allows for various phenomena such as superlensing, cloaking, and negative refractive index, enhancing many applications in medical imaging, telecommunications, and optics. This control of wave propagation becomes particularly important in the context of transmission line metamaterials. Their introduction within transmission line systems increases their performance at high frequencies, reduces losses, and enhances wave transmission. Nonlinear transmission line metamaterials do support such complex dynamical behaviors like pulse sharpening and soliton formation that result from nonlinear interactions involving variations in permittivity and permeability^1,2^. It is these features that directly support the wave dynamics and soliton behavior being investigated in this work.

More recently, interest in fractional-order models has been inspired by their attractive capabilities for modeling nonlocality, memory effects, and complex wave interactions more adequately than their classical integer-order models do^3–5^. These capabilities agree closely with metamaterial behavior in which dispersion, anomalous wave propagation, and nonlinear interactions are critical. In this context, fractional-order formulations have been applied to boundary value problems. Benchohra et al.^6^ derived existence results for boundary value problems involving nonlinear fractional differential equations and provided a mathematical foundation for the problems. Ahmad and Nieto^7^ examined the solvability of anti-periodic boundary value problems for fractional differential equations using the Leray–Schauder degree theory. Impulsive systems studied by Shah et al.^4^, who investigated the existence of positive solutions to a coupled system of impulsive boundary value problems with nonlinear fractional differential equations. Khan et al.^8–10^ contrasted the stability of nonlinear fractional differential equations with Caputo and Riemann–Liouville derivatives and differentiated their varying effects on system behavior. Moreover, they conducted a stability analysis and determined numerical solutions to a fractional-order HIV/AIDS model, illustrating the influence of fractional derivatives on epidemiological modeling. In 2020, the authors introduced a fractional-order model of HIV-TB coinfection under the nonsingular Mittag–Leffler law, offering insights into the disease dynamics. These researches validated the utilization of fractional calculus as a powerful tool in studying wave propagation in metamaterial-based transmission lines.

The conformable fractional derivative is important because it preserves the rules of differentiation; the chain rule is one of them, and because of this property, it is highly compatible with analytical soliton-generating techniques. Unlike Caputo or Riemann–Liouville derivatives, the conformable derivatives here have a greatly simplified and tractable formulation while modeling essential fractional-order effects that can be relevant to electromagnetic interactions. In the context of nonlinear wave propagation in metamaterial transmission lines, this derivative enables the governing partial differential equations to be reduced into solvable forms with the use of traveling wave transformations. This advantage allows obtaining explicit soliton solutions, supporting the paper’s focus on the analytical investigation of wave propagation.

Prior studies have introduced a wide range of relevant methods, including fractional Adams–Bashforth/Moulton and modified Euler schemes. Many fascinating methods were introduced. Zayernouri and Matzavinos^11^ suggested a fractional Adams–Bashforth/Moulton scheme for the Keller–Segel chemotaxis system, whereas Mazandarani and Kamyad^12^ presented a modified Euler scheme to fuzzy fractional initial value problems. Nazir et al.^13^ used a fractional-order approach for a COVID-19 model through a modified Euler scheme. Finite-difference and finite-element methods were employed by many authors. Abdulla et al.^14^ compared third-order fractional PDE solutions through explicit finite difference schemes. Additionally, Taha Abdulazeez and Modanli^15^ solved fractional pseudo-hyperbolic telegraph equations via finite difference schemes. There were other research studies that made use of finite element and volume approaches, e.g., Abedini et al.^16^, who proposed a Petrov–Galerkin approach for stochastic fractional equations, and Gao et al.^17^, who investigated space–time directional fractional diffusion equations. Likewise, Fang et al.^18^ and Jia and Wang^19^ introduced fast finite volume methods for fractional diffusion issues on nonuniform and locally refined grids, respectively.

Meshless and Fourier-based approaches were utilized by researchers like Du et al.^20^, where they carried out multi-term time-fractional integro-differential equations, and Lin et al.^21^, where they used a Fourier approach for (3 + 1)-dimensional fractional PDEs. Hilbert and wavelet-based approaches presented additional methods, such as Attia et al.^22^ and Abu Arqub^23^, where reproducing kernel Hilbert space methods were applied, and Faheem and Khan^24^ and Rabiei and Razzaghi^25^, where wavelet-based collocation and Boubaker wavelet approaches were used.

Feng^26^ introduced a novel approach employing the Jacobi elliptic equation in finding coefficient function solutions of conformable fractional partial differential equations. Fendzi-Donfack et al.^27^ constructed exotic soliton-like solutions for a fractional nonlinear electrical circuit equation by way of a differential-difference Jacobi elliptic functions methodology. Ali Akbar et al.^28^ explored soliton dynamics of the perturbed nonlinear Schrödinger equation and microtubules using an extended Kudryashov scheme. Ouahid et al.^29^ obtained new optical soliton solutions of the fractal-order Ginzburg–Landau equation using the generalized Kudryashov’s scheme. Duan et al.^30^ extended the adomian decomposition method with convergence acceleration to efficiently solve nonlinear fractional differential equations. Hu et al.^31^ derived analytical solutions of linear fractional differential equations by the adomian decomposition method. Farhood and Mohammed^32^ investigated time-fractional nonlinear variable-order delay partial differential equations using the homotopy perturbation method. Nawaz^33^ applied the variational iteration and homotopy perturbation methods for solving fourth-order fractional integro-differential equations. Wu^34^ derives a fractional variational iteration method for solving nonlinear fractional differential equations. Ibraheem et al.^35^ proposd an optimal variational iteration method to obtain the approximate solutions for fractional differential equations. Khan et al.^36^ found analytical solutions of fractional Klein-Gordon and gas dynamics equations using the (G′/G)-expansion method. Yao et al.^37^ solved conformable Drinfel’d–Sokolov–Wilson and Boiti-Leon-Pempinelli equations for exact solutions using the sine–cosine method. Fendzi-Donfack et al.^38^ constructed exotic solitons for a fractional circuit system using the sine–cosine method. Dubey and Chakraverty^39^ solved fractional coupled wave equations using a modified extended tanh method. Mamun et al.^40^ found exact traveling wave solutions of 3D fractional WBBM equations via an improved modified extended tanh-function method. Adem et al.^41^ analyzed solitary wave solutions of Fitzhugh-Nagumo-type equations with conformable derivatives.

Despite all these fascinating techniques, the similarity methods are still one of the common techniques in analyzing differential equations, either integer or fractional models^42–45^. In^42^, the authors discovered hidden symmetries and derived explicit solutions of the integro-differential Jaulent–Miodek evolution equation, contributing to research in integrable systems. In^43^, Lax pair and group transformation methods were applied to investigate integrability properties of the (3 + 1)-dimensional Boiti-Leon-Manna-Pempinelli equation. Kassem and Rashed^44^ analyzed N-soliton and cuspon wave solutions of the (2 + 1)-dimensional Broer-Kaup-Kupershmidt equations, and uncovered hidden symmetries within the framework of the Lie optimal system. New exact solutions were derived^45^ for the (3 + 1)-dimensional generalized Kadomtsev–Petviashvili equation by the combined use of Lie symmetry and singular manifold methods.

Yet, solitons are highly important to permit stable wave transmission, signal integrity, and high-precision telecommunications. Nonlinear transmission line metamaterials have, in essence, supported such localised wave structures that have offered applications in microwave engineering, high-frequency circuit design, and communication systems. This dynamic is enriched within fractional-order modeling because it allows additional control of dispersion and nonlinearity to understand wave interactions within metamaterials. Thus, the single and multiple structure soliton solutions obtained accordingly bear direct relevance in advanced waveguide design for enhanced signal flow, which was among the contributions described in the abstract. Younas et al.^46^ the paraxial nonlinear Schrödinger equation in Kerr media and derives diverse soliton and wave solutions using three advanced analytical techniques. The solutions are validated symbolically and illustrated through dynamic figures.

The novelty of this work consists in bringing together conformable fractional calculus with a nonlinear two-dimensional transmission line metamaterial model, which investigates soliton wave dynamics through several analytical schemes. Although various works have either been on classical-order transmission line models or specific fractional formulations, the present study is the first to apply and compare systematically three distinct analytical techniques, namely, the sine–Gordon expansion method, the fractional sub-equation method, and the tanh method within the very same conformable fractional framework. This unified treatment not only reveals new families of exact soliton solutions but also shows that the fractional-order parameter α can serve as a control mechanism for tuning wave propagation characteristics in engineered metamaterial structures. Consequently, the work offers a new route toward the design of next-generation fractional-order electromagnetic devices and an analytical toolbox versatile enough for exploring nonlinear dispersive behavior in transmission lines using metamaterials.

## Preliminaries and mathematical representation

This section introduces the fundamental concepts and notations that will be applied throughout the analysis. It provides a brief of relevant theoretical basics and definitions, and assumptions. The mathematical model of the problem is then established using appropriate symbols and equations.

### A Comparative analysis of conformable, non-conformable, Riemann–Liouville, and caputo fractional derivative

The core concept of fractional calculus can be briefed by two approaches. The first approach, known as the Riemann–Liouville approach, involves iteratively applying the integral operator $n$ times and then replacing it with a single integral using the Cauchy formula. In this process, the factorial notation $n!$ is replaced with the Gamma function, thereby defining the fractional integral of a non-integer order. Riemann and Caputo fractional derivatives are then defined using these integrals. The second approach, called the Grunwald–Letnikov approach, is based on iteratively applying the derivative operator n times and then using the Gamma function in the binomial coefficients to fractionalize it. The resulting fractional derivatives in this calculus may appear complex and lack certain fundamental properties of ordinary derivatives, such as the product rule and the chain rule. However, these fractional operators exhibit favorable semigroup properties in certain cases. Recently, some authors employed a new well-behaved fractional derivative known as the "conformable fractional derivative." This derivative is defined solely based on the basic limit definition of the derivative and offers simplicity and reliability^47–50^. Bilal et al.^47^ derived various exact optical soliton solutions for the generalized (2 + 1)-dimensional nonlinear conformable fractional Schrödinger system using three new integration techniques. The obtained solutions include dark, singular, combo, periodic, mixed, and plane wave solitons. Zulfiqar and Ahmed^48^ investigated new optical solutions of the conformable fractional perturbed Gerdjikov–Ivanov (pGI) equation using the tanh and tanh-coth methods. Ahmed et al.^49^ studied the conformable resonant nonlinear Schrödinger equation (CRNLSE) with Kerr law nonlinearity and derives new analytical solutions using the Exp-function, modified Exp-function. Muhammad et al.^50^ examined the complex dynamic behavior of the generalized fractional (2 + 1)-dimensional Bogoyavlensky–Konopelchenko equation, relevant to propagation phenomena using truncated M-fractional derivatives. The fractional order parameter strongly affects soliton stability and the bifurcation structure of the solutions. In practical metamaterial devices, adjusting this parameter may enable designers to tailor wave propagation properties, thereby improving performance in applications such as telecommunications and signal processing.

Consider a function $f:[0,\infty )\to {\mathbb{R}}$, the conformable fractional derivative of order $\alpha$ of $f$ is denoted by:

1 $${T}_{t}^{\alpha }\left(f\right)\left(t\right)=\underset{\varepsilon \to 0}{lim} \frac{f\left(t+\varepsilon {t}^{1-\alpha }\right)-f\left(t\right)}{\varepsilon }, \text{For all }t>0 \text{and }\alpha \in (\mathrm{0,1}),$$

properties of the conformable fractional derivative are as follows:

1. 2 $${T}_{t}^{\alpha }\left(af+bg\right)=a{T}_{t}^{\alpha }\left(f\right)+b{T}_{t}^{\alpha }\left(g\right), \text{for all }a,b\in {\mathbb{R}}$$
2. 3 $${T}_{t}^{\alpha }\left({t}^{p}\right)=p{t}^{p-\alpha }, \text{for all }p\in {\mathbb{R}}^{2}$$
3. 4 $${T}_{t}^{\alpha }\left(\uplambda \right)=0,\text{for all constant functions }f(t)=\lambda$$
4. 5 $${T}_{t}^{\alpha }(fg)=f{T}_{t}^{\alpha }(g)+g{T}_{t}^{\alpha }\left(f\right)$$
5. 6 $${T}_{t}^{\alpha }\left(\frac{f}{g}\right)=\frac{g{T}_{t}^{\alpha }(f)-f{T}_{t}^{\alpha }(g)}{{\mathrm{g}}^{2}}$$
6. 7 $${T}_{t}^{\alpha }\left(f\right)\left(t\right)={t}^{1-\alpha }\frac{df}{dt}\left(t\right), \text{where }f\text{ is differentiable}$$
7. 8 $${T}_{t}^{\alpha }(fog)(t)={t}^{1-\alpha }{g}^{{^{\prime}}}(t){f}^{{^{\prime}}}(g(t))$$

The conformable fractional derivative exhibits these characteristics, which are lacking in the Riemann–Liouville and Caputo fractional derivatives. It is important to note that the conformable fractional derivative is easier to define than the Riemann–Liouville and Caputo fractional derivatives. Two of the most important definitions are:

If n is a positive integer and α is in the range [n − 1, n], the derivative of N is:

- Riemann-Liouville definition:

9 $$D_{a}^{\alpha } \left( {\mathrm{M}} \right)\left( {\mathrm{t}} \right) = \frac{1}{{\Gamma \left( {{\mathrm{n~}} - {\mathrm{~}}\alpha } \right)}}\frac{{{\mathrm{d}}^{{\mathrm{n}}} }}{{dt^{n} }}\mathop \smallint \limits_{a}^{t} \frac{{{\mathrm{M}}\left( {\mathrm{u}} \right)}}{{\left( {{\mathrm{t~}} - {\mathrm{~u}}} \right)^{{\alpha - {\mathrm{n}} + 1}} }}du$$

- Caputo definition:

If n is a positive integer and α ∈ [n − 1, n), α derivative of N is given by.

10 $${D}_{a}^{\alpha }\left(\mathrm{M}\right)\left(\mathrm{t}\right)=\frac{1}{\Gamma \left(\text{n }-{ \alpha }\right)}{\int }_{a}^{t}\frac{{\mathrm{M}}^{\mathrm{n}}(u)}{{\left(\text{t }-\text{ u}\right)}^{{\alpha }-\mathrm{n}+1}}du$$

The conformable fractional derivatives provide a functional substitute for Riemann–Liouville and Caputo derivatives in the interval of 1/2 < α < 1. One of the best things about the conformable fractional derivative is how easy it is to define compared to the Riemann–Liouville and Caputo derivatives. The conformable fractional derivative is easier to understand and use than these typical fractional derivatives, which makes fractional calculus less complicated. The conformable fractional derivative also follows some of the basic rules of the regular derivative, like the product rule, quotient rule, and chain rule. These rules are not always followed by Riemann–Liouville and Caputo derivatives. This compliance with conventional derivative qualities makes it even more appealing and useful in a wide range of disciplines. The conformable fractional derivative also has a unique level of versatility because it lets you use both conformable and non-conformable kernels and change the order of α.

It is crucial to recognize that this study utilizes the conformable fractional derivative for its simplicity and retention of classical characteristics; however, alternative definitions, such as the Caputo or Riemann–Liouville derivatives, may influence the solvability and physical interpretation of the resultant wave equations. Subsequent study may examine these operators to enhance the validation and generalization of the current model’s conclusions. The conformable derivative is beneficial due to its simplicity; yet, it may not comprehensively capture the nonlocal and memory-dependent effects in nonlinear spatial systems. This may affect the accuracy of soliton forecasts at high frequencies.

### Fractional nonlinear electrical transmission lines equation

**Fig. 1:**
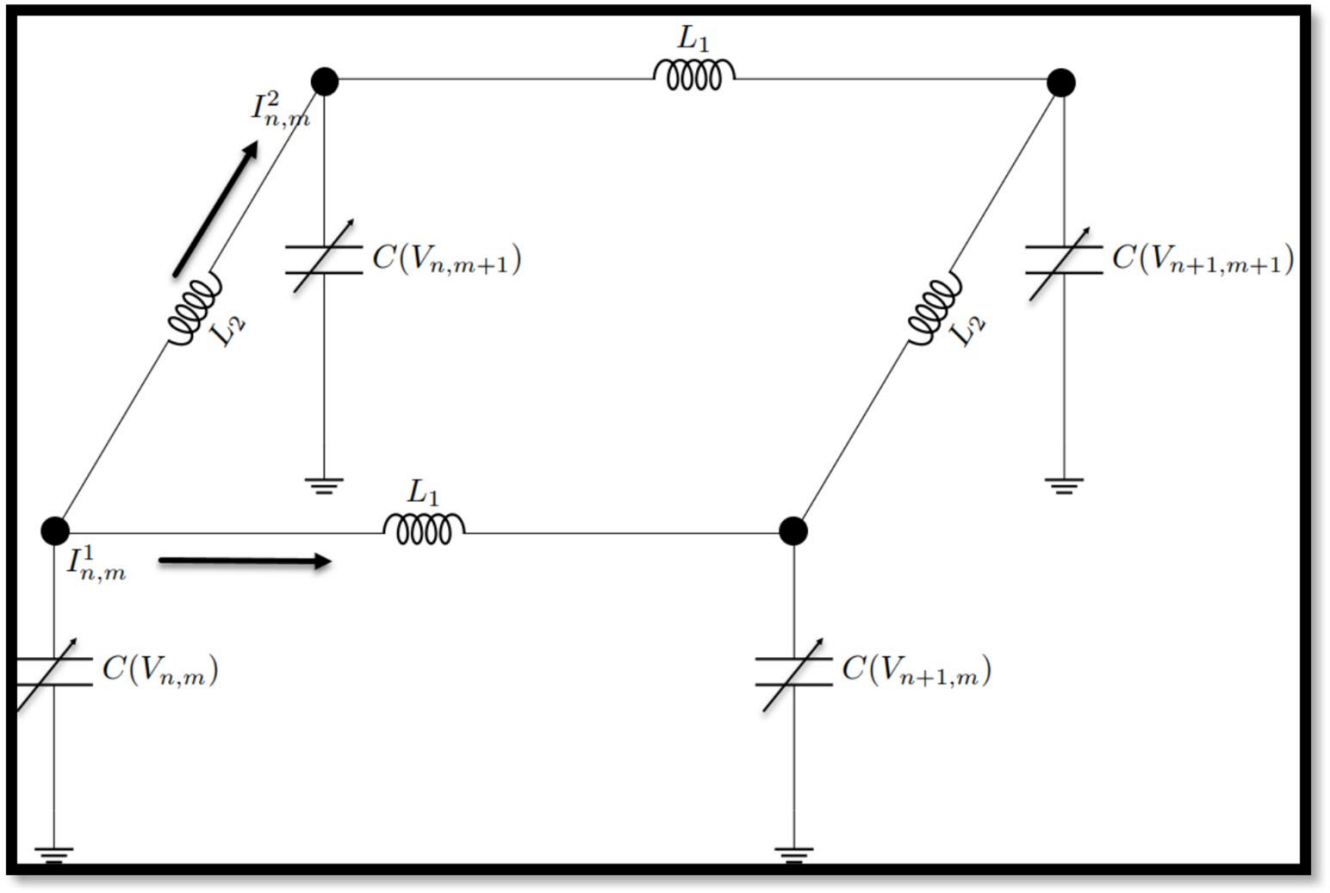
Schematic representation of the NCTL.

A network of a nonlinear electrical transmission line consists of coupled transmission lines with nonlinear circuit elements such as varactors or nonlinear inductors, generating complex wave dynamics. Unlike linear traditional transmission lines, these networks involve effects such as soliton generation, nonlinear dispersion, and wave modulation, thus being crucial in contemporary signal processing, power system stability analysis, and metamaterial studies. These nonlinear transmission networks could be employed in numerous applications including frequency conversion, pulse shaping, and optical communication. Wave behavior can be controlled by engineers to ensure maximum energy transfer and signal integrity in modern electronic and photonic systems through proper design of the topology and component characteristics^51^. Consider a network consisting of a periodic array of nodes connected by linear inductors ($L_{1} ,~L_{2}$) and nonlinear capacitors $C$($V$), arranged in a rectangular lattice configuration as depicted in Fig. 1.

The nonlinear capacitance used here varies according to the applied voltage as follows:

11 $$C\left({V}_{n}{,}_{m}\right)={C}_{0}\left(1-2a{V}_{n}{,}_{m}+3b{V}_{n}^{2}{,}_{m}\right)$$

where a and b are nonlinear coefficients that determine the electric charge stored in the capacitors, stratifying the Kirchhoff law on the above model, the following set of discrete differential equations are found:

12 $$\frac{{d}^{2}}{d{t}^{2}}\left({V}_{n}{,}_{m}-a{V}_{n}{,}_{m}+b{V}_{n}^{2}{,}_{m}+b{V}_{n}^{3}{,}_{m}\right)={u}_{0}^{2}\left({V}_{n+1}{,}_{m}+{V}_{n-1}{,}_{m-2}-2{V}_{n}{,}_{m}\right)+{\omega }_{0}^{2}\left({V}_{n}{,}_{m+1}+{V}_{n}{,}_{m-1}-2{V}_{n}{,}_{m}\right)$$

In this respect, Eq. (12) must be transformed into its continuum form^52^ and replace the linear derivative operators by the fractional time–space derivative of order $\alpha$.

13 $${D}_{t}^{2\alpha }\left(V-a{V}^{2}+b{V}^{3}\right)-{u}_{0}^{2}\left({\delta }_{1}^{2}{D}_{x }^{2\alpha }V+\frac{{\delta }_{1}^{4}}{12}{D}_{x }^{4\alpha }V\right)-{\omega }_{0}^{2}\left({\delta }_{2}^{2}{D}_{y }^{2\alpha }V+\frac{{\delta }_{2}^{4}}{12}{D}_{y }^{4\alpha }V\right)=0$$

Where

14 $${u}_{0}^{2}=\frac{1}{{L}_{1}{C}_{0}}, {\omega }_{0}^{2}=\frac{1}{{L}_{2}{C}_{0}}$$

To link the discrete transmission line model to continuous formulation, we assume that the lattice spacing is narrow enough to allow the difference equations to be replaced by differential equations. The normal derivatives are then expanded to fractional derivatives to better account for memory effects and unusual propagation phenomena. This change makes it possible to go from a discrete circuit representation to a continuous fractional partial differential equation model, making sure that the two methods are consistent with each other. Because inductors and capacitors are not evenly spaced out, the governing equations would need coefficients that depend on where they are in space. This would make them more complicated and maybe impossible to integrate. This puts lot of limits on analytical methods; hence numerical or perturbative methods may be needed^53^.

## Traveling wave solutions of the fractional nonlinear electrical transmission lines

In this section three distinct analytical methods namely the Sine–Gordon expansion, fractional sub-equation, and Tanh method is introduced and applied to derive explicit solutions for the model (13).

### Sine–Gordon expansion method

To understand the Sine–Gordon expansion (SGE) method, consider the fractional Sine–Gordon equation^54^:

15 $${T}_{x}^{2\beta }u-{T}_{t}^{2\alpha }u={m}^{2}\mathrm{sin}u$$

The traveling wave transformation is given in the form:

16 $$\xi =\mathrm{c}\frac{{t}^{\alpha }}{\alpha }+{K}_{1}\frac{{x}^{\alpha }}{\alpha }+{K}_{2}\frac{{y}^{\alpha }}{\alpha }, u\left(x,t,y\right)=U\left(\xi \right),$$

Inserting (16) into (15) reduces (15) to:

17 $${U}^{{^{\prime}}{^{\prime}}}=\frac{{m}^{2}\mathrm{sin}U}{{a}^{2}(1-{\nu }^{2})}$$

where ${\nu }^{2}=\frac{{b}^{2}}{{a}^{2}}$. Now, (17) can be written as:

18 $${\left(\frac{d\left(\frac{U}{2}\right)}{d\xi }\right)}^{2}=\frac{{m}^{2}}{{a}^{2}\left(1-{\nu }^{2}\right)}{\mathrm{sin}}^{2}\left(\frac{U}{2}\right)+c$$

where *c* is the constant of integration. Now, consider:

19 $$\omega \left(\xi \right)=\frac{U(\xi )}{2}, \frac{{m}^{2}}{{a}^{2}\left(1-{\nu }^{2}\right)}=1\text{ and }c=0$$

Then Eq. (18) becomes:

20 $$\frac{d\omega }{d\xi }=\mathrm{sin}\omega ,\text{ as }\omega =\omega \left(\xi \right)$$

The following relations are concluded from (20):

21 $$\mathrm{sin}\left(\omega \left(\xi \right)\right)=\frac{2C{e}^{\xi }}{{C}^{2}{e}^{2\xi }+1}={\left.\mathrm{sech}\xi \right|}_{C=1}$$

22 $$\mathrm{cos}\left(\omega \left(\xi \right)\right)=\frac{{C}^{2}{e}^{2\xi }-1}{{C}^{2}{e}^{2\xi }+1}={\left.\mathrm{tanh}\xi \right|}_{C=1}$$

According to the previous relations, the SGE method^54–56^ can be summarized as:

For any nonlinear fractional order partial differential equation:

23 $$p\left(u, {D}_{t}^{\alpha }u, {D}_{t}^{2\alpha }u,{D}_{x}^{\alpha }u ,{D}_{x}^{2\alpha }u,{D}_{y}^{\alpha }u,{D}_{y}^{2\alpha }u\dots \right)=0, 0<\alpha <1$$

where, *u* is the dependent variable and $p$ is a polynomial function in terms of $u$ and its derivatives. To find soliton solutions of (23) the method is performed through the following steps:

*Step 1:* Reduction of Eq. (23).

The fractional wave transformation (16) is firstly applied to reduce the (23) to an ordinary differential equation (ODE):

24 $$q\left(u,{u}^{{^{\prime}}},{u}^{{^{\prime}}{^{\prime}}},\dots \right)=0, u = u\left(\xi \right)$$

*Step 2:* The predicted solution of ODE (24) has the following form:

25 $$U\left(\xi \right)={A}_{0}+\sum_{j=1}^{s}{tanh}^{j-1}(\xi )({B}_{j}\mathit{sec}h\left(\xi \right)+{A}_{j}\mathit{tan}h\left(\xi \right))$$

which is expressed in $\omega$ as:

26 $$U\left(\omega \right)={A}_{0}+\sum_{j=1}^{s}{cos}^{j-1}(\omega )({B}_{j}\mathit{sin}\left(\omega \right)+{A}_{j}\mathit{cos}\left(\omega \right))$$

where* s* is a positive integer to be calculated throughout the balancing procedure between the nonlinear term in (24) and the highest derivative.

#### Applications of the method to NCTL model

The fractional wave transformation (16) reduces the NCTL (11) into an ODE in the form:

27 $${{c}^{2}D}_{\xi }^{2\alpha }\left(U-a{U}^{2}+b{U}^{3}\right)-{u}_{0}^{2}\left({\delta }_{1}^{2}{D}_{\xi }^{2\alpha }U+\frac{{\delta }_{1}^{4}}{12}{D}_{\xi }^{4\alpha }U\right)-{\omega }_{0}^{2}\left({\delta }_{2}^{2}{D}_{\xi }^{2\alpha }U+\frac{{\delta }_{2}^{4}}{12}{D}_{\xi }^{4\alpha }U\right)=0$$

Let ${K}_{1}={K}_{2}=K and {\delta }_{1}={\delta }_{2}=\delta ,$ Integrating twice yields the following equation:

28 $$-\frac{{K}^{4}{\delta }^{4}}{12}\left({u}_{0}^{2}+{\omega }_{0}^{2}\right){U}^{{\prime}{\prime}}+{c}^{2}\left(b{U}^{3}-a{U}^{2}\right)+({c}^{2}-{K}^{2}{\delta }^{2}\left(\left({u}_{0}^{2}+{\omega }_{0}^{2}\right)\right)U=0$$

Balancing the orders of the highest order derivative term with the highly nonlinear term (${U}^{{^{\prime}}{^{\prime}}}\&$ ${U}^{3}$) in (28), which can be considered as:

29 $$O\left[\left(\frac{{d}^{p}u}{{d\xi }^{p}}\right)\right]=S+p,\text{ and }O\left[{u}^{r}{\left(\frac{{d}^{q}u}{{d\xi }^{q}}\right)}^{m}\right]=Sr+m \left(q+S\right), \mathrm{respectively}$$

then we have ( $$S+2=3S)$$ leads to S = 1 and the predicted solution (26) takes the form:

30 $$U\left(\omega \right)={A}_{0}+{A}_{1}\mathrm{cos}\left(\omega \right)+{B}_{1}sin\left(\omega \right)$$

Substituting (30) into (28), considering (14), yields:

31 $$\begin{gathered} - \frac{{K^{4} \delta ^{4} }}{{12}}\left( {u_{0}^{2} + \omega _{0}^{2} } \right)( - B_{1} \sin ^{3} \left( \omega \right) + B_{1} \cos ^{2} \left( \omega \right)\sin \left( \omega \right) - 2A_{1} \cos \left( \omega \right)\sin ^{2} \left( \omega \right)) \hfill \\ + c^{2} \left( {b\left( {A_{0} + A_{1} \cos \left( \omega \right) + B_{1} sin\left( \omega \right)} \right)^{3} ~~~ - a~\left( {A_{0} + A_{1} \cos \left( \omega \right) + B_{1} sin\left( \omega \right)} \right)^{2} ~~~} \right) \hfill \\ + \left( {c^{2} - K^{2} \delta ^{2} \left( {\left( {u_{0}^{2} + \omega _{0}^{2} } \right)} \right)(A_{0} + A_{1} \cos \left( \omega \right) + B_{1} sin\left( \omega \right)} \right)~~~ = 0~.~ \hfill \\ \end{gathered}$$

Simplifying (31), then collecting the similar terms, and equating the coefficients to zero leads to the following system of algebraic equations:

32 $$\left\{\begin{array}{c}\frac{1}{12}{B}_{1}(12{c}^{2}b{B}_{1}^{2}+{K}^{4}{\delta }^{4}\left({\omega }_{0}^{2}+{u}_{0}^{2}\right))=0,\\ -\frac{1}{12}{B}_{1}(-36b{c}^{2}{A}_{1}^{2}+{K}^{4}{\delta }^{4}\left({\omega }_{0}^{2}+{u}_{0}^{2}\right))=0,\\ \frac{1}{6}{A}_{1}\left(18{c}^{2}b{B}_{1}^{2}+{K}^{4}{\delta }^{4}\left({\omega }_{0}^{2}+{u}_{0}^{2}\right)\right)+{A}_{1}^{3}b{c}^{2}=0,\\ -{c}^{2}{B}_{1}^{2}\left(a-3{A}_{0}b\right)=0,\\ \frac{-24}{12}{A}_{1}{c}^{2}{B}_{1}\left(-3{A}_{0}b+a\right)=0,\\ {c}^{2}\left(3{A}_{0}^{2}b-2{A}_{0}a+1\right)-{K}^{2}{\delta }^{2}\left({\omega }_{0}^{2}+{u}_{0}^{2}\right)=0,\\ -{A}_{1}{K}^{2}{\delta }^{2}\left({\omega }_{0}^{2}+{u}_{0}^{2}\right)+{A}_{1}{c}^{2}(-{A}_{1}^{2}b+3{A}_{0}^{2}b-2{A}_{0}a+1)=0,\\ {A}_{1}^{2}{c}^{2}(3{A}_{0} b-a)=0\\ -{A}_{0}{c}^{2}\left(-{A}_{0}^{2} b+{A}_{0}a-1\right)-{A}_{0}{K}^{2}{\delta }^{2}\left({\omega }_{0}^{2}+{u}_{0}^{2}\right)=0,\end{array}\right.$$

Solving the algebraic system, (32), results in:

$${A}_{0}=\frac{a}{3b} , {A}_{1}=0, {B}_{1}=i\frac{{K}^{2}{\delta }^{2}}{c}\sqrt{\frac{\left({\omega }_{0}^{2}+{u}_{0}^{2}\right)}{b}}, {B}_{1}=-i\frac{{K}^{2}{\delta }^{2}}{c}\sqrt{\frac{\left({\omega }_{0}^{2}+{u}_{0}^{2}\right)}{b}}$$ and

33 $$\xi =\mathrm{c}\frac{{t}^{\alpha }}{\alpha }+\frac{K}{\alpha }{(x}^{\alpha }{+y}^{\alpha }) .$$

34 $${u}_{1}\left(x,y,t\right)=\frac{a}{3b} + i\frac{{K}^{2}{\delta }^{2}}{c}\sqrt{\frac{\left({\omega }_{0}^{2}+{u}_{0}^{2}\right) }{b}} \mathrm{sech}(\mathrm{c}\frac{{t}^{\alpha }}{\alpha }+\frac{K}{\alpha }({x}^{\alpha }+{y}^{\alpha }))$$

35 $${u}_{2}\left(x,y,t\right)=\frac{a}{3b}- i\frac{{K}^{2}{\delta }^{2}}{c}\sqrt{\frac{\left({\omega }_{0}^{2}+{u}_{0}^{2}\right) }{b}} \mathrm{sech}(\mathrm{c}\frac{{t}^{\alpha }}{\alpha }+\frac{K}{\alpha }({x}^{\alpha }+{y}^{\alpha }))$$

**Fig. 2:**
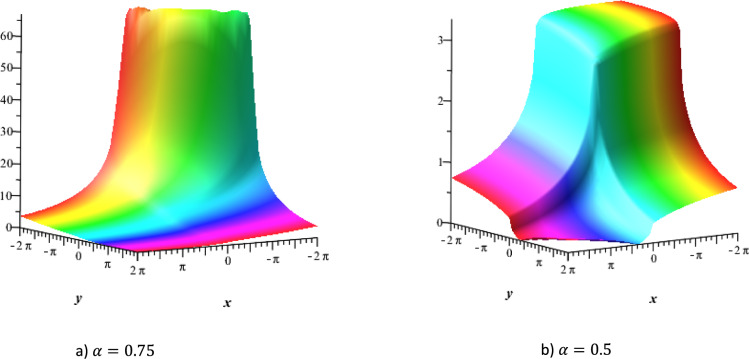
The kink soliton solution u_1_ with $\mathrm{a}=0.21,\mathrm{b}=0.197, t=1 {u}_{0}=3 , {\omega }_{0}=4,\delta =1,c=1 and K=0.3$.

**Fig. 3:**
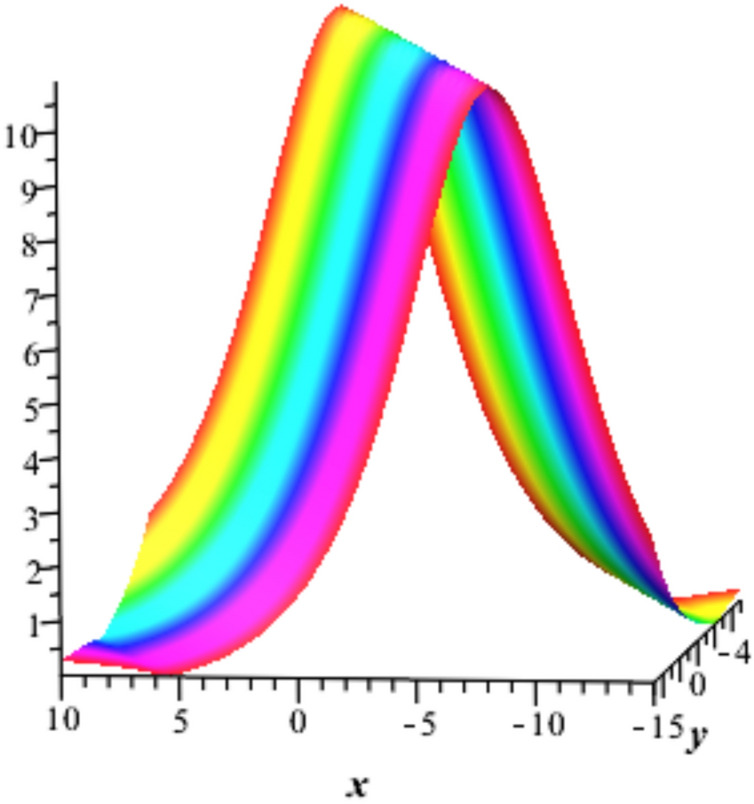
Bright soliton solution ${u}_{1}$ with $a=0.21,\text{ b}=0.197, t=1, {u}_{0}=3 , {\omega }_{0}=4,\delta =1,c=1,K=0.3\text{ and }\alpha =1$.

The solution ${u}_{1}$ is plotted for $\alpha =0.75$ and $\alpha =0.5$ in Fig. 2 (a)-(b) which represent three-dimensional profiles of typical nonlinear wave behaviors arising in NLTL due to the interplay of dispersion with Kerr-type nonlinearity. The first two show the localized wave packet with pronounced amplitude enhancement illustrating the bright-type and mixed bright-dark soliton structures that propagate stably along the line. Such profiles correspond to nonlinear voltage or current pulses generated in NLTLs incorporating nonlinear capacitive elements, where the balance between dispersion and cubic nonlinearity prevents waveform distortion^57^. Figure 3 depicts a strongly varying waveform with a shock-type or kink-shaped solution, which often arises when the line is subject to strong nonlinear steepening or discontinuities in its effective impedance. Such waveforms are indeed typical of the fast-rising pulses propagating in the strongly nonlinear ladder networks.

**Fig. 4:**
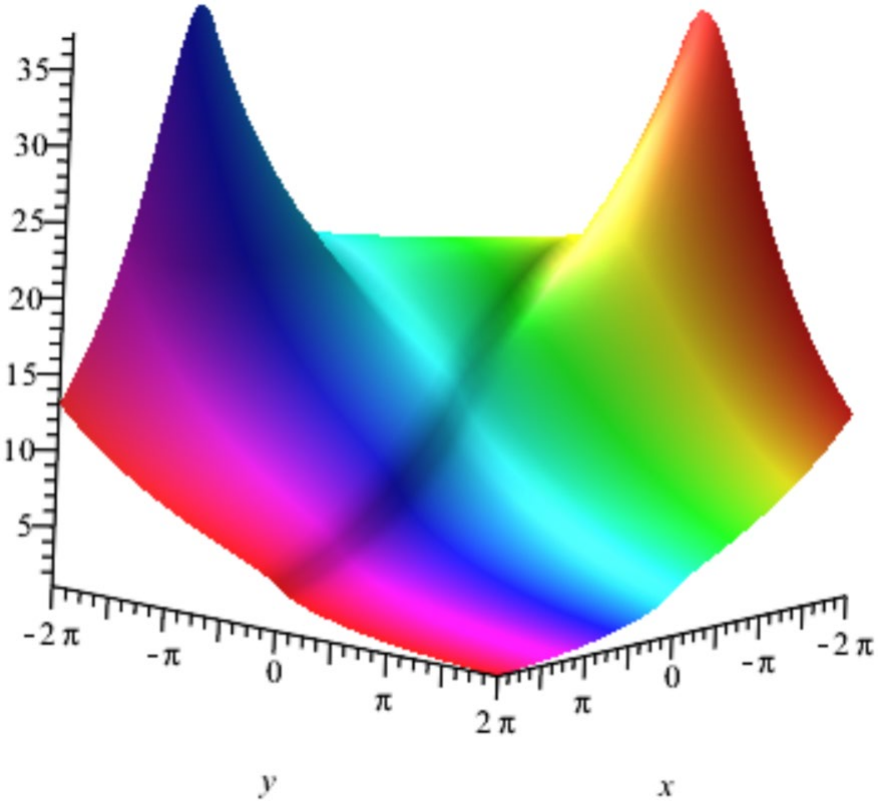
Lump soliton solution ${u}_{2}$ with ${a=0.5,b=0.6,t=1, u}_{0}=3 , {\omega }_{0}=4,\delta =1,c=0.6,K=0.3 and \alpha =0.75$.

The solution ${u}_{2}$ is plotted for $a=0.5,b=0.6,t=1$ ,${u}_{0}=3 , {\omega }_{0}=4,\delta =1,c=0.6,K=0.3 and \alpha =0.75$ in Fig. 4, represents a Lump soliton that describes the localized energy concentration with dual-peak structure, representing complex field distributions in two-dimensional metamaterial arrays that is physically applied in beam splitting and electromagnetic focusing devices.

### Tanh method

The tanh method is one of the most powerful techniques for analytical solutions of nonlinear evolution equations^58^. This method can be summarized in the following steps:

*Step 1:* Consider a nonlinear fractional partial differential, (23). First, the fractional traveling wave transformation (16) reduces (13) into an ODE in the form (24).

*Step 2:* A new independent variable *Y* is introduced:

36 $$Y =\text{ tanh }\left(\mu \xi \right).$$

The corresponding derivatives are then derived as follows:

37 $$\frac{du}{d\xi }=\frac{dY}{d\xi }\frac{du}{dY}=\mu sec{h}^{2}\left(\mu \xi \right)\frac{du}{dY}=\mu \left(1-tan{h}^{2}\left(\mu \xi \right)\right)\frac{du}{dY}=\mu \left(1-{Y}^{2}\right)\frac{du }{dY}$$

38 $$\frac{{d}^{2}u }{d{\xi }^{2}}=\frac{d}{d\xi }\frac{du}{d\xi }=\frac{d}{d\xi }\left(\mu \left(1-{Y}^{2}\right)\frac{du }{dY}\right)=\frac{d }{dY}\left(\mu \left(1-{Y}^{2}\right)\frac{du }{dY}\right)\frac{dY}{d\xi }= {\mu }^{2}{\left(1-{Y}^{2}\right)}^{2}\frac{{d}^{2}u }{d{Y}^{2}}-2{\mu }^{2}Y\left(1-{Y}^{2}\right)\frac{du }{dY}$$

39 $$\frac{{d}^{3}u}{d{\xi }^{3}}= 2{\mu }^{3}\left(1-{Y}^{2}\right)\left(3{Y}^{2}-1\right)\frac{du }{dY}-6{\mu }^{3}Y{\left(1-{Y}^{2}\right)}^{2}\frac{{d}^{2}u }{d{Y}^{2}}+{\mu }^{3}{\left(1-{Y}^{2}\right)}^{3}\frac{{d}^{3}u }{d{Y}^{3}}$$

and so on, where *μ* is a parameter that is to be determined.

*Step 3:* Assume the solution of (24) in the form of finite series expansion:

40 $$u\left(\xi \right)=S\left(Y\right)=\sum_{i=0}^{N}{a}_{i}{Y}^{i}.$$

The value of *N* will be calculated by balancing the highest order derivatives with the nonlinear terms in (24).

*Step 4:* Substituting Eqs. (37)-(39) into (24), using (40), then collecting the coefficients of like powers of ${Y}^{i}$, (*i* = 0, 1, 2, …), (24) is converted to a polynomial in $Y$. Equating each coefficient of the resulting polynomial to zero reveals system of algebraic equations, Solving the algebraic system with substituting the values of these constants ($\mu ,{K}_{1},{K}_{2},C$ ), obtain the exact solution of (24).

Prior to solving the reduced fractional ordinary differential equations, we remind ourselves that the standard theorems in fractional calculus (see Podlubny^59^ and Kilbas et al.^60^) ensure the existence and uniqueness of their solutions. Specifically, the obtained solutions are unique and exist if the nonlinear functions meet the boundedness and Lipschitz continuity conditions. The derived traveling wave solutions are mathematically well-posed since the nonlinearities in our reduced equations meet these requirements because they are polynomial in nature.

#### Applications of the method to NCTL model

As shown in the previous section the fractional wave transformation (16) reduces the NCTL, (13), into an ODE (28) with $N=1$. Then, Eq. (40) becomes.

41 $$u\left(\xi \right)=\sum_{i=0}^{1}{a}_{i}{Y}^{i}, u\left(\xi \right)={a}_{0}+{a}_{1}Y$$

Substituting (41) considering (36) in (24) results in:

42 $$\begin{gathered} \frac{{ - K^{4} \delta ^{4} }}{6}\left( {u_{0}^{2} + \omega _{0}^{2} } \right)\left( { - 2a_{1} \mu ^{2} Y + a_{1} \mu ^{2} Y^{3} } \right) \hfill \\ + c^{2} \left[ {\left( {ba_{0}^{3} + 2a_{0}^{2} a_{1} bY + 3a_{0} a_{1}^{2} bY^{2} + a_{1}^{3} bY^{3} } \right) - \left( {a_{0}^{2} a + 2a_{0} a_{1} aY + a_{1}^{2} aY^{2} } \right)} \right] \hfill \\ + c^{2} a_{0} + c^{2} a_{1} Y - K^{2} \delta ^{2} \left( {u_{0}^{2} + \omega _{0}^{2} } \right)\left( {a_{0} + a_{1} Y} \right) = 0 \hfill \\ \end{gathered}$$

Now, collecting the coefficients of ${Y}_{i},i=\mathrm{0,1},2,\dots$ and setting equal to zero, we obtain the following system:

43 $${Y}^{3}:\frac{-{K}^{4}{\delta }^{4}}{6}{a}_{1}{\mu }^{2}\left({u}_{0}^{2}+{\omega }_{0}^{2}\right)+{a}_{1}^{3}b{c}^{2}=0$$

44 $${Y}^{2}:3{a}_{0}{a}_{1}^{2}b{c}^{2}-a{a}_{1}^{2}{c}^{2}=0$$

45 $$Y:\frac{-{K}^{4}{\delta }^{4}}{6}{a}_{1}{\mu }^{2}\left({u}_{0}^{2}+{\omega }_{0}^{2}\right)+3{a}_{0}^{2}{a}_{1}b{c}^{2}-2{a}_{0}{a}_{1}a{c}^{2}+a1{c}^{2}-{K}^{2}{\delta }^{2}{a}_{1}\left({u}_{0}^{2}+{\omega }_{0}^{2}\right)=0$$

46 $${Y}^{0}:b{c}^{2}{a}_{0}^{3}-{a}_{0}^{2}a{c}^{2}+{a}_{0}{c}^{2}-{K}^{2}{\delta }^{2}{a}_{0}\left({u}_{0}^{2}+{\omega }_{0}^{2}\right)=0$$

Solving the algebraic system, results in:

47 $${a}_{0}=\frac{a}{3b} , {a}_{1}=\pm \frac{\mu {K}^{2}{\delta }^{2}}{c}\sqrt{\frac{\left({u}_{0}^{2}+{\omega }_{0}^{2}\right)}{6b}}$$

The exact solution for fractional nonlinear electrical transmission lines equation (NCTL).

48 $${u}_{3}\left(x,y,t\right)=\frac{a}{3b}+\left(\frac{\mu {K}^{2}{\delta }^{2}}{c}\sqrt{\frac{\left({u}_{0}^{2}+{\omega }_{0}^{2}\right)}{6b}}\right)\mathrm{tanh}\left(\mu \left(c\frac{{t}^{\alpha }}{\alpha }+\frac{K}{\alpha }({x}^{\alpha }+{y}^{\alpha })\right)\right)$$

49 $${u}_{4}\left(x,y,t\right)=\frac{a}{3b}-\left(\frac{\mu {K}^{2}{\delta }^{2}}{c}\sqrt{\frac{\left({u}_{0}^{2}+{\omega }_{0}^{2}\right)}{6b}}\right)\mathrm{tanh}\left(\mu \left(c\frac{{t}^{\alpha }}{\alpha }+\frac{K}{\alpha }({x}^{\alpha }+{y}^{\alpha })\right)\right)$$

**Fig. 5:**
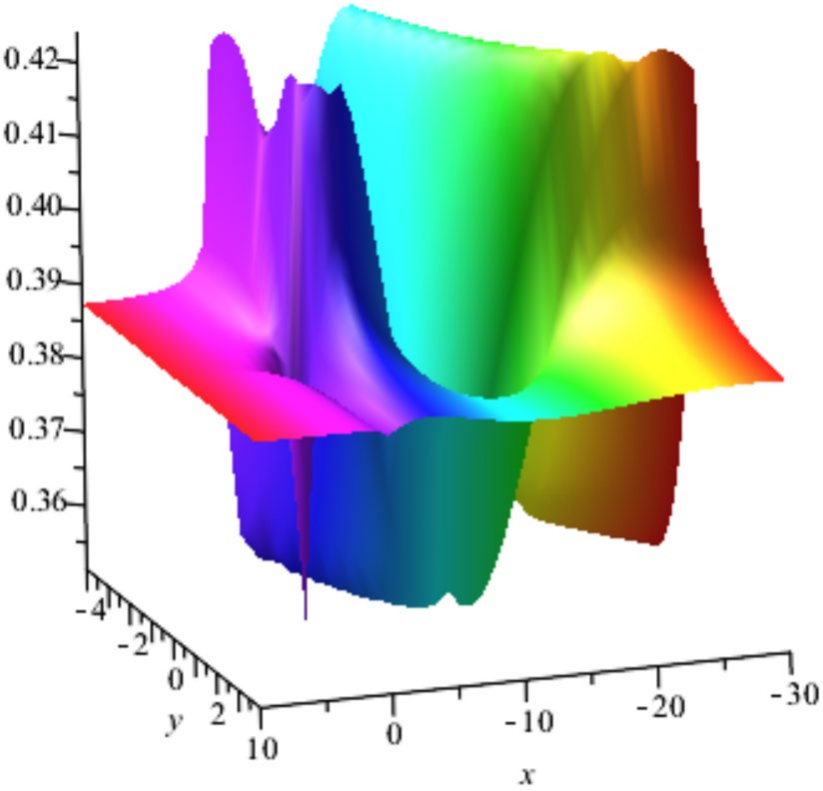
Rouge wave solution ${u}_{3}\left(x,y,t\right)$ with $a=0.5,b=0.19,t=3 , {u}_{0}=3 , {\omega }_{0}=4,\delta =1,c=0.5,K=0.3,\alpha =0.75 ,\mu =1.5$.

Figure 5 illustrates the Rogue wave solution in (48) at some specific parameters describing the sudden amplitude amplification phenomenon and explaining how nonlinear interactions in metamaterial transmission lines can lead to extreme wave events. This has important implications for device protection and high-power applications. The shown patterns reflect the ability of nonlinear transmission lines to break up an initially smooth waveform into several localized structures when subjected to high input energy or enhanced nonlinear coefficients. Such behavior characterizes the transition from stable soliton propagation to more complex or chaotic nonlinear dynamics.

### Fractional sub-equation method

In this section, the main steps of the fractional sub-equation method are described for solving fractional differential equations, which can be summarized in the following steps:

*Step 1:* Consider a nonlinear fractional partial differential, (23). First, the fractional traveling wave transformation (16) reduces (13) into an ODE in the form (24).

*Step 2:* According to the fractional sub-equation method, we assume that the solution of (24) can be expressed in the following finite series expansion form.

50 $$u\left(\xi \right)=\sum_{i=0}^{N}{a}_{i}{\left(\varphi \left(\xi \right)\right)}^{i}$$

where $ai\left(i =0, 1, 2, \dots ,N\right)$ are the constants to be determined later and $\varphi (\xi )$ satisfies the following fractional Riccati equation.

51 $${D}_{\xi }^{\alpha }\varphi \left(\xi \right)=\sigma +{\varphi }^{2}\left(\xi \right)$$

Where $\sigma$ is a constant.

*Step 3:* The value of *N* is determined through the homogeneous balance principle, then by employing the generalized Exp-function approach via Mittag–Leffler functions, first derived the following solutions of the fractional Riccati Eq. (51)

52 $$\varphi \left( \xi \right) = \left\{ {\begin{array}{*{20}c} { - \sqrt { - \sigma ~} tanh_{\alpha } \left( {\sqrt { - \sigma ~} ~\xi } \right),~~~~~~~~~~~~~~~~~~~~\sigma < 0,} \\ { - \sqrt { - \sigma ~} coth_{\alpha } \left( {\sqrt { - \sigma ~} ~\xi } \right),~~~~~~~~~~~~~~~~~~~~\sigma < 0,} \\ {\sqrt {\sigma ~} tan_{\alpha } \left( {\sqrt {\sigma ~} ~\xi } \right),~~~~~~~~~~~~~~~~~~~~~~~~~~~~~~\sigma > 0,~~} \\ { - \sqrt {\sigma ~} cot_{\alpha } \left( {\sqrt {\sigma ~} ~\xi } \right),~~~~~~~~~~~~~~~~~~~~~~~~~~~~~~~\sigma > 0,~~} \\ { - \frac{{\left( {1 + \alpha } \right)}}{{\xi ^{\alpha } + \omega }}~~~~,\omega = const~~~~~~~~~~~~~~~\sigma = 0.} \\ \end{array} ~~~~} \right.$$

where the generalized hyperbolic and trigonometric functions are defined as

$$sin{h}_{\alpha }\left(x\right)=\frac{{E}_{\alpha }\left({x}^{\alpha }\right)-{E}_{\alpha }\left(-{x}^{\alpha }\right)}{2} , cos{h}_{\alpha }\left(x\right)=\frac{{E}_{\alpha }\left({x}^{\alpha }\right)+{E}_{\alpha }\left(-{x}^{\alpha }\right)}{2} , tan{h}_{\alpha }\left(x\right)=\frac{sin{h}_{\alpha }\left(x\right)}{cos{h}_{\alpha }\left(x\right)}$$

53 $$cot{h}_{\alpha }=\frac{cos{h}_{\alpha }\left(x\right)}{sin{h}_{\alpha }\left(x\right)}, si{n}_{\alpha }\left(x\right)=\frac{{E}_{\alpha }\left(i{x}^{\alpha }\right)-{E}_{\alpha }\left(-i{x}^{\alpha }\right)}{2i },co{s}_{\alpha }\left(x\right)=\frac{{E}_{\alpha }\left(i{x}^{\alpha }\right)+{E}_{\alpha }\left(-i{x}^{\alpha }\right)}{2 }, \boldsymbol{ },\boldsymbol{ }ta{n}_{\alpha }=\frac{si{n}_{\alpha }\left(x\right)}{co{s}_{\alpha }\left(x\right)} ,co{t}_{\alpha }=\frac{co{s}_{\alpha }\left(x\right)}{si{n}_{\alpha }\left(x\right)}$$

And ${E}_{\alpha }(Z)$ denotes the Mittag–Leffler function, which is given as

54 $$E_{\alpha } \left( Z \right) = \mathop \sum \limits_{{i = 0}}^{\infty } \frac{{Z^{i} }}{{\Gamma \left( {i\alpha } \right)}}$$

*Step 4:* Substituting (50) along with (51) into (24) and using the properties of the conformable fractional derivative. A polynomial in $\varphi (\xi )$ could be obtained. Setting all the coefficients of ${\varphi }_{i} (i = 0, \mathrm{1,2}, \dots ,)$ to zero yields a set of nonlinear algebraic equations in ${a}_{i}(i = 0, 1, 2, \dots , N), k, c and \sigma .$

*Step 5:* By solving the nonlinear algebraic equations obtained in step 4, we get the values of the constants. Substituting these constants and the solution of (52) into (24), we can obtain the explicit solutions of (23).

Now consider the fractional sub-equation method to obtain the exact solution for NCTL, which is previously reduced to the ODE (28), with N = 1,

Then Eq. (50) can be written in the form:

55 $$u\left(\xi \right)={a}_{0}+{a}_{1}\varphi \left(\xi \right).$$

Substituting (55) and (51) in (24) results in:

56 $$\begin{gathered} - \frac{{K^{4} \delta ^{4} }}{6}\left( {u_{0}^{2} + \omega _{0}^{2} } \right)\left( {a_{1} \sigma \varphi + a_{1} \varphi ^{3} } \right) \hfill \\ + c^{2} \left[ {ba_{0}^{3} + 3a_{0}^{2} a_{1} ~\varphi + 3a_{0} a_{1}^{2} \varphi ^{2} + a_{1}^{3} ~\varphi ^{3} } \right) - a\left( {a_{0}^{2} + 2a_{0} a_{1} ~\varphi + a_{1}^{2} \varphi ^{2} } \right)] \hfill \\ + c^{2} \left( {~a_{0} + a_{1} \varphi } \right) - K^{2} \delta ^{2} ~\left( {u_{0}^{2} + \omega _{0}^{2} } \right)\left( {a_{0} + a_{1} \varphi } \right) = 0 \hfill \\ \end{gathered}$$

Now, collecting the coefficients of ${\varphi }_{i},i=\mathrm{0,1},2,\dots$ and setting these to zero, we.

Obtain the following system:

57 $${\varphi }^{0}:{a}_{0}^{3}{c}^{2}b-a{a}_{0}^{2}{c}^{2}+{a}_{0}{c}^{2}-{\mathrm{K}}^{2}{\updelta }^{2}{\mathrm{a}}_{0} \left({\mathrm{u}}_{0}^{2}+{\upomega }_{0}^{2}\right)=0$$

58 $$\varphi :-\frac{{\mathrm{K}}^{4}{\updelta }^{4}}{6}\left({\mathrm{u}}_{0}^{2}+{\upomega }_{0}^{2}\right){a}_{1}\sigma +3{c}^{2}b{a}_{0}^{2}{a}_{1}-2{a}_{0}{a}_{1}a{c}^{2}+{a}_{1}{c}^{2}-{\mathrm{K}}^{2}{\updelta }^{2} \left({\mathrm{u}}_{0}^{2}+{\upomega }_{0}^{2}\right){a}_{1}=0$$

59 $${\varphi }^{2}: 3{a}_{0}{a}_{1}^{2}{c}^{2}b-a{a}_{1}^{2}{c}^{2}=0$$

60 $${\varphi }^{3}: -\frac{{\mathrm{K}}^{4}{\updelta }^{4}}{6}\left({\mathrm{u}}_{0}^{2}+{\upomega }_{0}^{2}\right){a}_{1}+{a}_{1}^{3}{c}^{2}b= 0$$

Solving the algebraic system, results in:

61 $${a}_{0}=\frac{a}{3b} , {a}_{1}=\pm \frac{{\mathrm{K}}^{2}{\updelta }^{2}}{c\sqrt{6}}\sqrt{\frac{\left({\mathrm{u}}_{0}^{2}+{\upomega }_{0}^{2}\right)}{b}} , \sigma =\frac{-6{c}^{2}\left({a}^{2}-3b\right)}{3b{\mathrm{K}}^{4}{\updelta }^{4}\left({\mathrm{u}}_{0}^{2}+{\upomega }_{0}^{2}\right)} -\frac{6}{{\mathrm{K}}^{2}{\updelta }^{2}}$$

62 $$u\left(\xi \right)=\frac{a}{3b}\pm \frac{{\mathrm{K}}^{2}{\updelta }^{2}}{c\sqrt{6}}\sqrt{\frac{\left({\mathrm{u}}_{0}^{2}+{\upomega }_{0}^{2}\right)}{b}}\varphi \left(\xi \right)$$

Then the exact solution for fractional nonlinear electrical transmission lines equation (NCTL).

63 $${u}_{\mathrm{1,2}}\left(x,y,t\right)=\frac{a}{3b}\pm \frac{{K}^{2}{\delta }^{2}}{c\sqrt{6}}\sqrt{\frac{\left({u}_{0}^{2}+{\omega }_{0}^{2}\right)}{b}}(-\sqrt{-\sigma }tan{h}_{\alpha }\left(\sqrt{-\sigma } \left(c\frac{{t}^{\alpha }}{\alpha }+\frac{K}{\alpha }({x}^{\alpha }+{y}^{\alpha })\right)\right)$$

64 $${u}_{\mathrm{3,4}}\left(x,y,t\right)=\frac{a}{3b}\pm \frac{{K}^{2}{\delta }^{2}}{c\sqrt{6}}\sqrt{\frac{\left({u}_{0}^{2}+{\omega }_{0}^{2}\right)}{b}}(-\sqrt{-\sigma }cot{h}_{\alpha }\left(\sqrt{-\sigma } \left(c\frac{{t}^{\alpha }}{\alpha }+\frac{K}{\alpha }({x}^{\alpha }+{y}^{\alpha })\right)\right)$$

The parametric dependencies resulting from each formulation were compared compared in order to examine the amplitude and speed of the soliton solutions obtained using various analytical techniques. Whereas the tanh and sub-equation approaches result in more limited profiles, the Sine–Gordon expansion typically produces solutions with modifiable amplitude-speed relations. The scaling laws show that the Sine–Gordon method captures a wider range of physically realistic soliton behaviors under fractional dynamics assumptions, especially when matching parameters to metamaterial transmission line data. This comparison demonstrates each method’s predictive power under particular analytical limitations.

By adding higher-order nonlinearities to the current–voltage relations, a nonlinear inductive term, either in addition to or instead of a nonlinear capacitor, would alter the governing fractional transmission line equations. This modification affects the form and solvability of traveling wave solutions by shifting the ratio of dispersion to nonlinearity. Specifically, it could change the threshold conditions for the existence of soliton solutions or give rise to new classes of them. According to the analysis, nonlinear inductance broadens the model’s physical applicability to a larger class of metamaterial devices by enriching the solution space.

To delineate the validity of the closed-form solutions, we non-dimensionalize the model and identify the triplet of control parameters (α, χ, δ) corresponding to fractional order, nonlinearity, and dispersion, respectively. The Sine–Gordon expansion is valid for $0.6\le \alpha \le 1$ and the amplitude remains within the weak-to-moderate regime. The tanh-ansatz requires monotone profiles and fails for oscillatory/multi-peak states or for α ≪ 1 where memory effects dominate. The sub-equation approach admits solutions only when the balancing condition between the highest-order nonlinear and dispersive terms is satisfied. In all cases physical admissibility is enforced by ${l}_{eff}>0$, ${C}_{eff}>0$, bounded constitutive laws, and non-negative energy density. Outside these windows, the formulae may become non-physical or cease to converge.

## Conclusion

This study performed a detailed analysis of the soliton solutions of the nonlinear conformable fractional-order transmission line metamaterials model (NCTL) by applying three remarkably efficient analytical approaches: Sine–Gordon expansion (SGE), fractional sub-equation, and Tanh methods. The findings are to develop the theory and application of wave propagation, nonlinear dispersion, and soliton interactions in electrical transmission line metamaterials. The following have been attained.

1. The applied fractional traveling wave transformation successfully reduced the nonlinear partial differential equation (PDE) model to ordinary differential equations (ODEs) in order to possess explicit soliton solutions.
2. The derived soliton and multi-soliton solutions demonstrate increased insight into energy transfer and signal integrity in nonlinear electrical transmission systems.
3. The solutions confirm the fractional-order derivatives role to incorporate memory effects and nonlocal interactions, as required by emerging microwave circuits and telecommunication systems.
4. The Sine–Gordon Expansion Method (SGE) demonstrated enhanced generality and adaptability in generating solutions that may be tailored for various metamaterial configurations. The method produces more generalized forms than the Tanh and fractional sub-equation methods, yielding a wider array of soliton solutions with variable parameters. This versatility enables the modeling of various wave patterns in nonlinear transmission lines. These generalized forms are particularly advantageous for practical applications since they enhance the efficacy and adaptability of future telecommunications systems by facilitating the creation of intricate metamaterial structures.
5. The Tanh and fractional sub-equation methods provided complementary information on soliton interactions, pulse shaping, and energy distribution.
6. The fractional NCTL model preserves wave dispersion, nonlinear energy localization, and soliton stability, which provides valuable insights into signal processing, power electronics, and high-frequency transmission systems.
7. The conformable fractional derivative is defined more simply than the Riemann–Liouville and Caputo fractional derivatives.
8. The solitary wave solution is not only mathematically significant but also possesses physical and engineering relevance. The correlation among wave speed, stability, and energy concentration indicates possible applications in practical systems, including telecommunications and signal processing.
9. The precise soliton solutions derived herein presuppose the maintenance of spatial symmetry. Changes in symmetry might affect the existence or shape of solutions, which could lead to changes in wave structures or limits on the validity of solutions. This constraint underscores the necessity for additional exploration into conditions that disrupt symmetry.

## Future prospects

The incorporation of external forcing terms and higher-dimensional fractional models would additionally refine the insight into nonlinear wave interactions in complicated transmission networks. Moreover, the proposed soliton solutions can find applications in multi-layer transmission systems and intricate photonic circuits, paving the way for communication technologies of the future.

- If the nonlinear components of the transmission line equations were introduced involving memory-dependent constitutive rules, the ensuing soliton propagation would probably exhibit prolonged interaction effects and more intricate wave phenomena. Future endeavors will focus on this extension to enhance the realism of metamaterial dynamics depiction. Future research may improve the analytical framework by combining the effects between electric and magnetic field dynamics in anisotropic metamaterials. This kind of generalization would make the model more physically accurate and make it more useful for real-world devices.
- Made it clear that a piecewise or discontinuous capacitance relation can cause sudden changes in soliton behavior and change the structure of the solution. Future research may tackle this issue by hybrid analytical–numerical methodologies.
- It was noted that substituting the conformable time derivative with a spatially variable-order derivative would enable the model to encapsulate graded material reactions and heterogeneity; however, this necessitates sophisticated methodologies and is suggested for future investigation.

## Data Availability

The data that support the findings of this study are available within the article.
